# Mechanisms of Post-Pancreatitis Diabetes Mellitus and Cystic Fibrosis-Related Diabetes: A Review of Preclinical Studies

**DOI:** 10.3389/fendo.2021.715043

**Published:** 2021-09-10

**Authors:** Eleonóra Gál, Jurij Dolenšek, Andraž Stožer, László Czakó, Attila Ébert, Viktória Venglovecz

**Affiliations:** ^1^Department of Pharmacology and Pharmacotherapy, University of Szeged, Szeged, Hungary; ^2^Faculty of Medicine, University of Maribor, Maribor, Slovenia; ^3^Faculty of Natural Sciences and Mathematics, University of Maribor, Maribor, Slovenia; ^4^First Department of Medicine, University of Szeged, Szeged, Hungary

**Keywords:** diabetes of the exocrine pancreas (DEP), acute pancreatitis (AP), chronic pancreatitis (CP), cystic fibrosis (CF), interaction

## Abstract

Anatomical proximity and functional correlations between the exocrine and endocrine pancreas warrant reciprocal effects between the two parts. Inflammatory diseases of the exocrine pancreas, such as acute or chronic pancreatitis, or the presence of cystic fibrosis disrupt endocrine function, resulting in diabetes of the exocrine pancreas. Although novel mechanisms are being increasingly identified, the intra- and intercellular pathways regulating exocrine–endocrine interactions are still not fully understood, making the development of new and more effective therapies difficult. Therefore, this review sought to accumulate current knowledge regarding the pathogenesis of diabetes in acute and chronic pancreatitis, as well as cystic fibrosis.

## Introduction

The pancreas is a unique organ, having both the endocrine and exocrine functions. The exocrine pancreas is composed of acini, which are dome-shaped clusters of acinar cells that produce and secrete enzymes involved in the digestion of food. Enzymes are secreted into the ductal tree that is formed by another cell type, the $\text{HC}O_{3}^{-}$-producing ductal cells that neutralise low pH from the stomach and provide an optimal environment for the enzymes to operate. Unlike the exocrine component, the endocrine pancreas produces hormones that enter the bloodstream and regulate carbohydrate metabolism. There is ever-accumulating evidence supporting the existence of a strong functional interrelationship between the exocrine and endocrine pancreas, and the repercussion of endocrine dysfunction in the exocrine function and vice versa (1–5). Therefore, understanding exocrine–endocrine interactions is crucial for the diagnosis and treatment of pancreatic diseases. Diabetes of the exocrine pancreas (DEP) develops secondary to exocrine pancreatic disorder. One of the most common diseases of the exocrine pancreas is acute and chronic pancreatitis (AP and CP, respectively). AP is a sudden inflammation of the pancreas, whereas CP is a persistent condition that arises from repeated damage of the pancreas and is associated with fibrosis, calcification and destruction of the gland. In both forms of pancreatitis, the risk of developing diabetes is high (6, 7); however, the underlying mechanism is not completely known. In addition to pancreatitis, cystic fibrosis (CF) is also often associated with diabetes, especially at advanced age (8–10). Although CF is not specifically an inflammatory disease, the secretory defect due to the dysfunction of the cystic fibrosis transmembrane conductance regulator (CFTR) Cl^-^ channel causes inflammation and fibrosis of the pancreas that can also affect the endocrine functions.

Recently, DEP has been increasingly emphasized in clinical practice (11–14), although concerns remain regarding its treatment. Compared to type 1 (T1DM) and type 2 (T2DM) diabetes mellitus (DM), the pathogenesis of DEP has been relatively less researched, with most experimental data focusing on CF. Considering the lower incidence of DEP compared to the other two types of diabetes and the limited data available, diagnosing DEP remains difficult. In addition, no specific guidelines have been established for the treatment of DEP, although more and more recommendations have recently emerged (3, 15–18). Two recent reviews provided a detailed and comprehensive analysis of the current diagnostic and treatment guidelines for DEP and recommended the use of a novel nomenclature (12, 14) Accordingly, the classification of DEP includes new-onset diabetes after pancreatitis, i.e., post-pancreatitis diabetes mellitus (PPDM); pancreatic cancer-related diabetes; and CF-related diabetes (CFRD). Based on this, diabetes after AP should be termed PPDM-A, whereas diabetes after CP should be termed PPDM-C.

This review sought to accumulate current experimental knowledge regarding the endocrine–exocrine interactions, focusing on PPDM-A, PPDM-C, and CFRD.

## Effect of the Exocrine Pancreas on Endocrine Function Under Physiological Conditions

Since the discovery of the “insulo-acinar axis” in early 1960s (19), the impact of insulin on pancreatic exocrine function has been extensively studied both *in vivo* and *in vitro* and these studies indicated that insulin has a significant effect on exocrine function (20–27). In contrast, much less information is available regarding the effect of exocrine cells on the endocrine function. In pigs, oral pancreatic enzyme pretreatment was shown to decrease plasma insulin levels for intravenous glucose tolerance test (GTT) and test meal (28). The same workgroup demonstrated that in exocrine pancreatic insufficient pigs, supplementation of exocrine enzymes reduced plasma insulin levels after both starvation and oral or intravenous GTT (29). Similarly, alpha-amylase supplementation also reduced plasma insulin levels after both intravenous and duodenal GTT, while it increased glucagon levels several folds in pigs with T1DM and T2DM (30). The inhibitory effect of amylase on insulin secretion has also been demonstrated in the insulin-producing cell line, BRIN-BD11 (30), and in hamsters with induced peripheral insulin resistance (IR) (31–33). In addition to amylase, lipase also plays an important role in insulin secretion by reducing the formation of long-chain fatty acids, which regulate glucose-induced insulin secretion through the activation of G-protein-coupled receptor, GPR40 (32, 34). These data indicate that amylase and lipase not only play a role in the breakdown of carbohydrates and fats but also directly or indirectly inhibit insulin release after ingestion of large amounts of carbohydrates or fats. The purpose of the inhibitory effect of amylase and lipase may be to keep insulin at a normal level; however, the exact significance of this is not entirely clear. One possible explanation is a regulatory mechanism that acts against insulin overproduction and thus exhaustion of beta cells. Among the proteases, chymotrypsin does not affect either insulin secretion, islet size, or the number of beta cells; however, this has only been demonstrated in hamsters and no other studies have been conducted to confirm these results in humans (32). In contrast, intracellular trypsin significantly increases the activity of K_ATP_ and reactivates the channel after complete rundown by modifying the regulatory protein of K_ATP_ or the channel itself (35, 36). Opening of the channel causes K^+^ efflux and, as a result, hyperpolarization of the membrane, which in turn inhibits voltage-gated Ca^2+^ channels and exocytosis of insulin granules. The physiological significance of the inhibitory effect of trypsin on insulin secretion is not fully understood, but it is likely to play an important role in normalizing serum insulin levels after a meal, such as amylase and lipase. Beside the regulation of insulin secretion, trypsin also plays a role in islet formation and differentiation through PAR-2 receptor activation and calcium signalling (37).

## Effect of the Exocrine Pancreas on Endocrine Function Under Pathophysiological Conditions

Under certain pathological conditions, the secretion of digestive enzymes is altered. Inflammation of the pancreas destroys the parenchyma, and prolonged inflammation leads to the development of exocrine pancreatic insufficiency, a condition in which the production of pancreatic enzymes is greatly reduced. Due to decreased enzyme production, insulin secretion is released from the inhibitory effect of digestive enzymes that leads to hyperinsulinemia. Clinical studies have demonstrated that serum insulin level increases after AP and CP, although in both cases, hyperinsulinemia is explained by the decreased insulin clearance, and not by the overproduction of insulin (38, 39). Regardless of the cause of hyperinsulinemia, it is unclear whether this transiently high insulin level plays a role in DEP. Previous studies have demonstrated that hyperinsulinemia may be responsible for the development of IR (40–42), a condition in which cells become less sensitive to insulin. Because IR is considered as a precursor of diabetes, hyperinsulinemia during AP and CP may be a sign of subsequent DEP development. The following chapters describe the mechanisms that are thought to play a role in the development of DEP in AP, CP, and CF.

### Post-Acute Pancreatitis Diabetes Mellitus

AP is an inflammatory disorder of the pancreas and one of the most frequent reasons for hospitalisation related to a gastrointestinal condition (43). AP can progress rapidly and cause severe symptoms like systemic inflammatory response, which may lead to multi-outcome and predisposes patients to diabetes. Research regarding the incidence of post-acute pancreatitis diabetes mellitus **(**PPDM-A), as well as factors associated therewith, has remained controversial. A recently published systematic review and meta-analysis that examined the incidence of new-onset DM after AP based on various criteria, including the severity of pancreatitis, aetiological factors, presence of necrosis and follow-up duration (7), showed a 23% overall incidence of DM after AP. Although similar results had been found by Das et al. (1), other workgroups showed much higher (44–47) or lower incidences (48, 49). Severity of pancreatitis, aetiology and the presence of necrosis have also been considered the most important risk factors for the development of diabetes (7). With regard to severity, some studies found no correlation between the development of diabetes and severity of pancreatitis (1, 44, 50–53), whereas others found a strong association between them, with the extent of necrosis being a decisive factor (44, 47, 51, 54, 55). Generally, the number of functionally active beta cells decreases as the extent of necrosis increases, leading to altered insulin secretion. Research regarding aetiological factors has been inconsistent. Majority of the studies indicate that diabetes is most often associated with alcohol-induced pancreatitis (7, 55), which may be partly explained by the increased incidence of diabetes among patients with alcoholic and biliary AP in such studies. With regard to other aetiological factors, hyperlipidaemia-induced pancreatitis has been found to cause diabetes in more patients (86%) compared to the other aetiologies (53). In addition, prior to the onset of diabetes, patients with AP often develop IR. Obesity is a risk factor in the development of IR (56, 57). Accumulated adipose tissue releases a number of inflammatory mediators that can affect insulin receptor or insulin binding to the receptor through various signalling pathways. Consistent with this, it has been reported that obesity increases the risk of IR development in patients with AP (58). Furthermore, in non-obese AP patients, increased intra-pancreatic fat deposition was associated with an increased HOMA-IR (homeostasis model assessment-IR) index (59). Among the inflammatory mediators, the adipocytokine IL-6 has been shown to be associated with elevated levels of chronic hyperglycaemia and IR after AP (60). This proinflammatory cytokine is released from adipose tissue and can inhibit both the insulin receptor and the action of insulin (61). Upregulation of IL-6 has been also shown in experimental models of AP where it is associated with enhanced local and systemic response. The association between IR and AP severity was investigated in a prospective clinical study, where IR was shown to be an independent factor in predicting AP severity (62). These data suggest that in addition to increasing the risk of diabetes development, IR also exacerbates the outcome of pancreatitis.

Experimental studies on rats have shown that sodium taurocholate-induced pancreatitis did not alter islet morphology or GLUT-2 expression but reduced insulin secretion in response to glucose stimulation, indicating functional deterioration of beta cells (63). Similar results were obtained in an l-arginine-induced rat model wherein no change in alpha and beta cell counts but a significant decrease in insulin secretion was observed 1 month after the induction of pancreatitis (64). Kinami et al. also found no morphological abnormalities in islet cells after the induction of AP but did observe a significant decrease in the serum levels of insulin and glucagon (65). Interestingly, they found that damage occurred earlier in alpha cells than beta cells. The exact mechanism that leads to a decrease in hormone production after AP is not completely known. Experiments in rats with acute necrotizing pancreatitis have shown that endoplasmic reticulum stress and nitric oxide production play significant roles in beta cell dysfunction (66–68), although other factors are presumably also involved. Our workgroup had also investigated the morphology and function of the endocrine pancreas after caerulein-induced AP in mice (*unpublished data*). Accordingly, we found that mice with caerulein-induced AP showed lower fasting insulin levels in the acute inflammation phase but higher fasting glucagon levels compared to the untreated control group. Fasting blood glucose levels were nonetheless normal. Intraperitoneal glucose tolerance tests in the same animals showed normal response to glucose by both hormones but significantly lower insulin levels in the caerulein-treated group. Blood glucose levels were normal and did not differ in caerulein-treated *versus* control mice. Immunofluorescent staining of whole pancreas tissue sections against insulin and glucagon did not reveal significant differences in islet morphology between the caerulein-treated and control groups. The aforementioned results suggest that the high levels of tissue necrosis observed in AP do not significantly affect islet morphology or islet cell counts but significantly alter serum hormone levels, particularly the level of insulin, which presumably leads to the deterioration of metabolism observed in patients with AP.

Furthermore, there is growing evidence that the exocrine pancreas and endocrine pancreas interact with each other not only through their secretions but also indirectly, through dysbiosis, resulting from inflammation. Sun et al. have shown that the level of an antimicrobial peptide, cathelicidin-related antimicrobial peptide (CRAMP), changes during inflammation (69). This peptide is produced by beta cells and plays an important role in shaping the immune environment of the pancreas. Production of CRAMP is regulated by short-chain fatty acids (SCFAs) which are produced by the gut microbiota. Since AP is associated with dysbiosis, the production of SCFAs and thus the level of CRAMP decreases during AP, which may lead to the development of T1D through the formation of an unfavourable immune environment (69). These data suggest that the development of diabetes in AP is a highly complex process, in which—besides the direct effect of the inflammatory environment in the pancreas—other indirect factors also play a role.

### Post-Chronic Pancreatitis Diabetes Mellitus

CP is a progressive disease characterised by parenchymal destruction, as well as the presence of inflammation and fibrosis. Heavy alcohol consumption has been the most common cause of CP worldwide, although other factors, such as genetic mutations, hypertriglyceridemia, hypercalcaemia and pancreatic duct obstruction, may also play a role. According to the necrosis–fibrosis hypothesis, recurrent acute attacks on the pancreas cause irreversible damage of the exocrine pancreas and lead to the development of CP (70). Loss of islet cells and the development of hepatic IR are often associated with long-standing CP, which results in diabetes among such patients (71). CP is typically considered to be one of the major causes of DEP (72). Around 30% of CP patients develop diabetes, although such rates can reach as high as 90% depending on the follow-up duration and region (6, 73, 74). Alcoholism and distal pancreatectomy have been considered the most common independent risk factors for DEP in CP (75–77), while male sex, steatorrhea, and biliary stricture have also emerged as risk factors for diabetes development, with divided opinions regarding smoking (73, 74). Only a limited number of guidelines are available for the diagnosis and treatment of DEP in CP. The most accepted and currently used guideline was developed by the HaPanEU/United European Gastroenterology, which contains specific diagnostic and therapeutic recommendations (78). According to current recommendations, therapy should include treatment of hyperglycaemia, steatorrhea and malnutrition. Although recent efforts have been made to improve treatment, such patients still have higher mortality rates compared to those with CP alone, with the presence of diabetes considerably impairing their quality of life. Therefore, comprehensive knowledge regarding the pathomechanism of PPDM-C would definitely promote substantial improvements in therapy.

A number of experiments have been conducted to identify intra- and extracellular mechanisms related to the development of diabetes in CP. Most of these studies have used the long-term partial pancreatic duct ligation (PDL) technique for the induction of CP that causes extensive acinar cell damage and tissue fibrosis. According to the most widely accepted view, the major cause of impaired metabolism or diabetes in CP is defective insulin secretion (79–81). As the disease progresses, the pancreas becomes more fibrotic, which impedes proper blood supply to the islets, causing progressive loss of beta cells and impaired islet function. However, alterations in glucose metabolism have also been observed not only during disease progression but also during the early stages, which predisposes patients to the development of diabetes (82). In a clinical cohort, patients with CP who had no diabetes and pancreatic calcification showed higher fasting and mixed-meal glucose levels and lower insulin sensitivity compared to healthy controls (82). However, those with advanced CP without diabetes exhibited reduced beta cell mass, and the expression of pancreatic and duodenal homeobox gene as well as insulin gene decreased in these patients (83). Recent research has shown that the decrease in beta cell number among patients with CP was not due to cell death, but rather the epithelial–mesenchymal transition (EMT) of beta cells (84). Xiao et al. demonstrated that high levels of transforming growth factor β1 (TGF-β1) regulates EMT through the SMAD3/Stat3 signalling pathway. This hypothesis is supported by previous studies showing that TGF-β1 overexpression promotes massive fibrosis, abnormal islet distribution and the appearance of fibroblast-like cells and macrophages (85). Furthermore, the Stat3 antagonist forkhead box protein O1 has been shown to prevent the development of EMT, as well as diabetes. Furthermore, the inflammatory milieu resulting from pancreatitis also contributes to beta cell dysfunction. Several inflammatory mediators have been shown to inhibit insulin release (IL-1β, IFN-γ) or to be associated with IR (IL-6) (86–88). The accumulation of IFN-γ within the islets is presumably due to the differentiation of Th17 cells into IFN-γ-producing Th1 cells by the inflammatory environment (89). Under physiological conditions, insulin inhibits gluconeogenesis and glycogenolysis in the liver and promotes glycogen synthesis (90). Andersen et al. had shown that the inhibitory effect of insulin on hepatic glucose production is mediated by the reduction in hepatic GLUT-2 receptors (91). However, given the lack of sufficient insulin in cases with CP, no such reduction was observed, while GLUT-2 internalisation was inadequate (91, 92). In addition, they showed that insulin receptors interact with GLUT-2 transporters, a mechanism that allows insulin to regulate hepatic glucose transport (93). Apart from insulin, other islet hormones, such as PP, also have decreased secretion in CP, which can play a significant role in altered glucose metabolism (94). Several studies have revealed that PP cells on the periphery of the islets protect beta cells in the centre, while decreased PP production may be a sign of reduced insulin production and can be used to predict the development of diabetes (95–97). Bastidas et al. who characterised the effect of PP infusion on glucose tolerance and insulin response in CP dogs found that intravenous administration of PP did not improve glucose tolerance or insulin response (98) but did increase hepatic sensitivity to insulin. CP decreases the number of insulin receptors on hepatocytes, which probably plays role in the development of hepatic IR (99). Exogenous administration of PP increases the expression of hepatic insulin receptors, as well as the binding of insulin to its receptor (99, 100), thereby enhancing the sensitivity of hepatocytes to insulin (100). Studies have shown that intravenous administration of PP restored hepatic IR in rats, dogs and humans with CP (101–103), which could suggest the therapeutic use of PP. Banerjee et al. showed that packaging of PP into micelles increases its half-life and thus its efficacy (104). A randomised clinical trial investigating the effects of PP on insulin requirements in 10 patients with T1DM or DEP found that subcutaneous administration of PP reduced insulin requirements in these patients (105). In contrast to decreased hepatic insulin sensitivity, peripheral insulin sensitivity increases in CP, which may be explained by an increase in the number of insulin receptors on blood cells and an increase in insulin binding to its receptors (106, 107). However, activated inflammatory cells and cytokines present systemically in CP may play a role in the development of IR. Immune cells in the peripheral blood of CP patients show elevated expression of the cytokines, IL-2, IL-6, IL-12 and IFN-γ, and decreased expression of IL-4 and IL-10, indicating an increased inflammatory state (108). Similarly, plasma levels of IL-6, TNF-α and adiponectin are significantly elevated in CP (109). Chronic low-grade inflammation has long been considered to promote the development of IR (110). TNF-α is an adipose tissue-derived proinflammatory cytokine that causes IR by enhancing adipocyte lipolysis and increasing the serine/threonine phosphorylation of insulin receptor substrate-1 (IRS-1) through the JNK and IKKβ/NF-κB pathways (110–113). IFN-γ is suggested to promote IR by inhibiting insulin action and adiponectin secretion in adipocytes (114) and was shown to inhibit glucose-stimulated insulin response in CP (87). IL-6 is also recognised as an inflammatory mediator that causes IR by reducing the expression of GLUT4 and IRS-1 by activating the Janus kinase-signal transducer and activator of transcription (JAK-STAT) signalling pathway and increasing suppressor of cytokine signalling 3 (SOCS3) expression (115, 116). Through activation of STAT3, IL-6 can also lead to IR in skeletal muscle by inducing the expression of toll-like receptor-4 (TLR-4), which is suggested to be a major upstream molecule in the activation of NF-κB. Furthermore, IL-6 is also found to induce IR by impairing the synthesis of glycogen through downregulating the expression of miR-200s and upregulating that of FOG-2 (56, 117–119). Cytokines released from adipose tissue may therefore damage the insulin responsiveness also of skeletal muscle and further exacerbate IR in CP. Intramuscular adipose tissue content has been shown to affect the disease severity and survival rate in patients with pancreatic diseases (120, 121). IR in CP is exacerbated by obesity, which is a common concomitant condition in CP patients. In fact, obesity can be characterised as a state of chronic low-grade inflammation, promoting IR (122, 123). Clinical studies show that the duration of DM in CP patients correlates positively with BMI and obese patients are more likely to develop severe AP with a more intense systemic inflammatory response (124–126).

**Figure 1:**
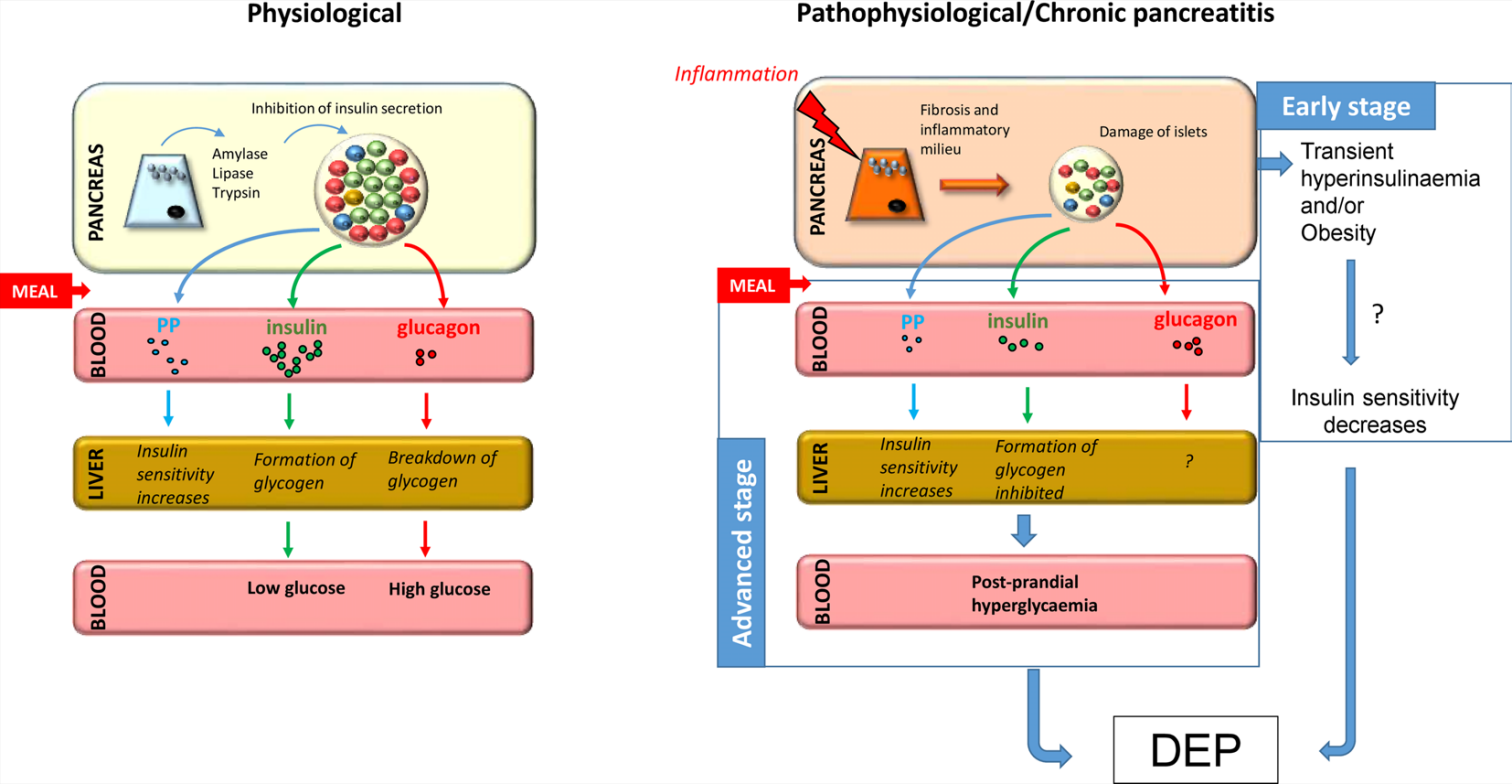
Putative mechanism for the development of DEP in CP. Under normal conditions, food ingestion triggers secretion of enzymes from acinar tissue, insulin from beta cells, and pancreatic polypeptide from PP cells and inhibits secretion of glucagon from alpha cells. In chronic pancreatitis, fibrosis and inflammatory milieu lead to destruction of islets and decreased hormone production, especially insulin and pancreatic polypeptide, resulting in decreased glycogen formation and increased hepatic IR, respectively. Taken together, this leads to post-prandial hyperglycaemia and predisposes patients to the development of diabetes at advanced stages. Furthermore, the early stage of the disease is often associated with hyperinsulinemia, which together with obesity, may play a role in the development of IR, which is a known precursor to diabetes.

Serum glucagon levels can also change during CP. A previous study by Donowitz et al. involving 10 patients with CP found that CP reduced basal glucagon levels, while the infusion of l-alanine, an endogenous stimulator of glucagon secretion, was unable to enhance glucagon response (127). Moreover, basal blood glucose levels in these patients were higher compared to controls, indicating that glycaemic control is disturbed in CP. Similar results were found in another study that compared patients with T2DM to those who developed PPDM-C (128). This study found that both the diabetes and CP groups had higher serum glucose levels after OGTT compared to controls and the serum glucagon level also showed an initial increase in these groups, which is presumably due to the fact that postprandial glucagon release is not inhibited due to decreased endogenous insulin production. Interestingly, this increase in glucagon levels was not observed during intravenous GTT, suggesting that other intestinal hormones, such as incretin hormones, may also play a role in abnormal glucagon levels (129). In contrast, glucagon levels decreased under hypoglycaemic conditions. Previous studies have also observed low levels of glucagon during hypoglycaemia in patients with pancreatitis, with the rate of decrease being directly proportional to the stage of the disease (130, 131). Defective alpha cell function in diabetes and CP can be partly explained by damage to most of the beta cells, which render them unable to properly control the function of alpha cells (128). In addition, a recent study on a patient with advanced CP who had previously undergone partial pancreatectomy found that the number of alpha cells significantly increased mostly around middle-size ducts and in the lumen of the ducts, suggesting that newly formed alpha cells during regeneration may have ductal origin or that ductal cells play a substantial role in islet neogenesis (132). **Figure 1** shows a possible mechanism for the development of DEP in CP.

### Cystic Fibrosis-Related Diabetes

CF, the most common genetic disorder in Caucasian populations with a prevalence of 1 out of 3,500 individuals in the United States (133), is caused by mutations in the CFTR gene (134). Approximately, 2,000 *CFTR* gene mutations have been identified and classified into six groups depending on their biological effects (135). F508del, the most common mutation, causes an abnormal folding of the CFTR protein, which consequently degrades immediately after synthesis or does not function as a Cl^−^ channel even when it reaches the cell membrane. The CFTR channel is located at the apical membrane of secretory epithelial cells and mediates the transport of Cl^−^ and $\text{HC}O_{3}^{-}$ ions (136). The lungs and pancreas are the most affected organs in patients with CF. In the lungs, CFTR plays an essential role in the formation of airway surface liquid (ASL) (137) that lines the airway epithelium and ensures mucociliary clearance (138). Owing to the decreased Cl^−^ secretion and increased Na^+^ absorption in CF, the volume and composition of ASL change, leading to airway surface dehydration and decreased mucociliary transport that favours airway infections (139). Persistent infections induce consecutive inflammation and greatly impairs respiratory function, ultimately leading to the death of patients with CF (140). The pancreas of patients with CF displays extensive fibrosis, fat infiltration and significant loss of islets (50%) (141). In the pancreas, CFTR is expressed on ductal cells and with the $CI^{-}/\text{HC}O_{3}^{-}$ exchanger, responsible for the secretion of $\text{HC}O_{3}^{-}$ -rich pancreatic fluid (136). Defects in $\text{HC}O_{3}^{-}$ secretion increase the viscosity of the pancreatic fluid, which favours the formation of mucus plugs in the ductal tree and leads to premature activation of digestive enzymes, resulting in the destruction of the pancreas (142).

The introduction of novel therapies has considerably improved the survival of patients with CF in recent years. Currently, the median predicted survival age of patients with CF is 41–46 years, depending on the type of mutations and the gender of the patients (143, 144). A retrospective cohort analysis had shown that females have worse survival than males (144), for which the female hormone, oestrogen, is at least partly responsible. Oestrogen increases the incidence of the mucoid form of *Pseudomonas aeruginosa* in the lungs, which shows greater resistance to antibiotics and thus induces more severe inflammation (145). Nevertheless, the increased age of patients with CF has also been associated with an increased incidence of comorbidities. The most common comorbidity associated with CF is CF-related diabetes (CFRD) (8), a multifactorial disease that, although present more frequently in advanced age, can develop at any age (146). Over the age of 40, 83% of women and 64% of men with CF have been found to develop diabetes (147). Moreover, a strong correlation exists between the type of CFTR mutation and the development of diabetes. Patients with mild, class IV or class V mutations are less likely to develop diabetes than those with more severe, class I or class II mutations (147). Those with class I, II and III mutations exhibited significantly greater impairment in pancreatic exocrine functions compared to those with class IV and V mutations (148), which explains why diabetes is more common among class I and II mutations (147). The presence of diabetes substantially reduces the life expectancy of patients with CF (149), especially women (147, 150). Lewis et al. had shown that patients with CFRD had a 3.5 times higher mortality rate compared to those with CF (147). The shorter life expectancy among patients with CFRD can be mainly attributed to accelerated deterioration of lung function due to continuous bacterial infections. Hyperglycaemia in these patients provides energy for bacteria and promotes their growth (151). Furthermore, patients with CFRD present with poor nutritional status and microvascular complications (nephropathy, retinopathy and neuropathy) (9).

Diabetes in patients with CF is not considered T1DM or T2DM. Basically, severe damage to the exocrine pancreas destroys beta cells, resulting in decreased insulin secretion and therefore the development of CFRD. However, the exact molecular background for the development of diabetes is not fully elucidated in CF, partly due to the controversial role of CFTR on beta cells. One of the main reasons for this controversy is that CFTR is differentially expressed in different species and has various endocrine functions. Using isolated islets or a pancreatic beta cell line, mouse islets have been shown to express functionally active CFTR (152–156). Studies using genetically (F508del) or pharmacologically (CFTRinh172) developed CF mice have demonstrated characteristic changes in the morphology of the islets in the absence of CFTR (155, 156). Accordingly, although the size and insulin content of the islets decreased significantly, the cell number remained unchanged. In addition, centralisation of alpha cells can be observed. In both CF models, a non-hyperglycaemia-associated decrease in pancreatic and serum levels of insulin had been noted. Pharmacological inhibition of CFTR did not significantly affect serum glucagon levels but did increase it several folds in F508del mice (155, 156). The importance of CFTR in insulin secretion has also been confirmed in a mouse insulinoma cell line, MIN6, where genetic or pharmacological inhibition of CFTR caused a reduction in insulin secretion that was further decreased in the presence of oxidative stress (154). Guo et al. had shown that CFTR activation in beta cells is required for membrane depolarisation and calcium mobilisation associated with insulin secretion. They also demonstrated that incubation of F508del beta cells with the VX-809 corrector (Lumacaftor) dose-dependently increased insulin secretion (153). Nonetheless, oral administration of the CFTR corrector and activator (Lumacaftor and Ivacaftor) to patients with CF who have the F508del mutation did not improve glucose tolerance or insulin secretion (157, 158). In contrast to mice, Boom et al. found that CFTR was mainly localised to alpha cells in rat pancreatic islets (159). In the ferret CF model, the size and composition of islets show a dynamic change as the disease progresses (160). A comparison of CF ferrets at different stages showed that islet sizes and the number of alpha, beta and gamma cells increased significantly at more advanced stages. One study also demonstrated that the increased islet cell count presumably results from the transdifferentiation of ductal cells into islet cells, in which matrix metalloproteinase-7, a remodelling factor, plays an essential role (160).

Regarding the human pancreas, White et al. had recently shown that less than 1% of normal adult beta cells express CFTR (161). Further studies have indicated that insulin secretion defects in CFRD are not due to the lack of intrinsic CFTR function (162, 163). Hart et al. had shown that the absence of CFTR did not affect alpha and beta cell function and CFRD development much more related to beta cell loss and inflammatory cell infiltration (162). Moreover, Sun et al. found that CFTR regulates beta cells through pro-inflammatory factors released from exocrine cells (163). However, regardless of species, it is generally accepted that exocrine inflammation in CF damages the entire pancreas to such an extent that the number of beta cells and thus insulin secretion are considerably reduced (162). However, this hypothesis somewhat contradicts the fact that the remaining beta cells must produce enough insulin to prevent the development of diabetes, which suggests that CFTR intrinsically regulates insulin secretion and that the functional defect in residual beta cells causes the development of CFRD. Nevertheless, the development of CFRD with age suggests that the pancreas suffers progressive damage wherein a continuous decline of islet cells can be observed. The role of age in the pathomechanism of CFRD is also shown by the fact that insulin sensitivity also decreases over time in CF patients with glucose abnormalities (164). Unlike beta cells, however, much less information is available on the role of CFTR in alpha cells. In pancreatic alpha cells, CFTR inhibits glucagon secretion (165–167), presumably by stimulating K_ATP_ channels (167). Patients with CF exhibit dysregulated glucagon secretion, which probably also contributes to abnormalities in their glucose tolerance.

## Conclusion

Considering that the exocrine pancreas and endocrine pancreas affect each other’s function through a number of pathways, it is not surprising to observe endocrine dysfunction in cases with exocrine insufficiency. During acute or chronic inflammation of the exocrine pancreas, the development of diabetes is highly dependent on the severity of the disease, whereas in CF, the development of diabetes has been found to increase with the length of the disease. However, all tree diseases are characterized by dysregulated hormone secretion. Given that early detection of disturbances in glucose homeostasis can prevent the development of more serious complications, monitoring carbohydrate metabolism in these patients is strongly recommended, as well as establishing appropriate diagnostic criteria of prime importance considering that DEP is often misdiagnosed as T2DM. After proper diagnosis of DEP, appropriate treatment of the disease constitutes another problem. Owing to the yet fully elucidated pathomechanism of DEP, developing specific therapies has remained difficult. Currently, treatment of DEP has been based on guidelines specific for the treatment of T2DM. Therefore, establishing guidelines that differentiate DEP according to not only other types of diabetes but also the different aetiologies thereof is greatly needed. Furthermore, there needs to be a strong emphasis on basic research considering that better understanding of the pathomechanism of the disease can substantially contribute to the identification of novel therapeutic targets.

## Author Contributions

All authors listed have made a substantial, direct, and intellectual contribution to the work and approved it for publication.

## Funding

This study was supported by the CFRD-SRC Grant (No. SRC 007), the National Research, Development and Innovation Office (SNN134497 to VV and K128222 to LC) grants, the New National Excellence Program of the Ministry of Human Capacities (UNKP-18-4), and the Slovenian Research Agency (research core funding nos. P3-0396 and I0-0029, as well as research projects nos. J3-9289, J4-9302, J1-9112, N3-0048, N3-0133, and N3-0170).

## Conflict of Interest

The authors declare that the research was conducted in the absence of any commercial or financial relationships that could be construed as a potential conflict of interest.

## Publisher’s Note

All claims expressed in this article are solely those of the authors and do not necessarily represent those of their affiliated organizations, or those of the publisher, the editors and the reviewers. Any product that may be evaluated in this article, or claim that may be made by its manufacturer, is not guaranteed or endorsed by the publisher.
